# Predictive characterization and molecular docking of lignocellulolytic enzymes: comparative analyses of Moroccan fungal species

**DOI:** 10.5114/bta/221148

**Published:** 2026-08-20

**Authors:** Soukaina Arif, Mouna Janati, Mohamed Benaddou, Said Oulghazi, Hassan Hajjaj

**Affiliations:** 1 Laboratory of Biotechnology and Bioresources Valorization, Faculty of Sciences, Moulay Ismail University, BP 11201 Zitoune Meknes, Morocco; 2 Laboratory of Biodiversity, Ecology and Genome, Plant and Microbial Biotechnology, Biodiversity and Environment Research Centre, Faculty of Sciences, Mohammed V University, BP 1014 RP, Rabat, Morocco

**Keywords:** Moroccan fungi, lignocellulose degradation, β-glucosidase, enzyme modeling, molecular docking

## Abstract

**Background:**

Lignocellulosic biomass has significant potential as a renewable feedstock for the production of biofuels and bioproducts. However, its structural complexity, particularly the crystalline nature of cellulose and protective lignin matrix, poses considerable challenges for enzymatic degradation. Fungi isolated from diverse lignocellulosic wastes, particularly olive pomace in Morocco, are a significant, yet underutilized resource of lignocellulolytic enzymes with a potential to overcome these challenges.

**Materials and methods:**

This study investigated the structural and functional properties of lignocellulolytic enzymes derived from 9 filamentous fungi. A total of 80 curated sequences were systematically categorized into cellulases and ligninases. Phylogenetic analysis was conducted on representative endoglucanases, β-glucosidases, and laccases to assess their diversity. Physicochemical parameters, secondary structure content, and thermostability indices were determined using ExPASy ProtParam and SOPMA. Homology models were generated with SWISS-MODEL and validated through PROCHECK, ERRAT, and ProSA. Molecular docking with AutoDock was used to evaluate the interactions of β-glucosidase with cellobiose.

**Results:**

Phylogenetic analysis revealed high evolutionary diversity among the examined species. Several enzymes showed favorable aliphatic indices and GRAVY scores, suggesting thermostability and hydrophilicity. β-Glucosidase from *Fusarium equiseti* showed the strongest predicted binding energy to cellobiose (–5.32 kcal/mol), with hydrogen bonding mediated by key residues, including Asp88, Arg94, Lys185, Gln197, Asp276, and Gln278, at optimal distances (1.95–2.58 Å).

**Conclusions:**

This integrative *in silico* study highlights the predictive potential of Moroccan fungal β-glucosidases, particularly from *F. equiseti*, for future applications in lignocellulose bioconversion.

## Introduction

Lignocellulosic biomass has attracted considerable attention in recent studies because of its high renewable energy content, cost-effectiveness, and carbon neutrality (Jahangeer et al. 2024). Lignocellulose is a highly complex structure involving a cellulose-based core scaffold, surrounded by a matrix of hemicellulose and lignin (Barhoum et al. 2020). Cellulose, the most abundant polysaccharide in nature, is considered a promising renewable resource for producing alternative fuels. However, its enzymatic degradation is challenging owing to its crystalline structure and the presence of lignin, which limits accessibility to cellulolytic enzymes. Enzymes involved in lignocellulose degradation belong to the broader group of carbohydrate-active enzymes (CAZymes), whose classification is primarily based on bioinformatic analyses of amino acid sequence similarity, structural features, and catalytic mechanisms. This systematic framework established by the CAZy database categorizes enzymes into families that reflect evolutionary relationships and functional specialization (Lombard et al. 2014). In this classification scheme, fungal cellulolytic systems are primarily composed of glycoside hydrolases (GHs) that act synergistically to hydrolyze β-1,4-glycosidic bonds in cellulose; this enzyme activity is complemented by oxidative auxiliary activities (AAs) that enhance substrate accessibility. Classical cellulases include endoglucanases (EC 3.2.1.4; GH5, GH6, GH7, GH9, GH12, GH45, and GH74), which randomly cleave internal bonds in amorphous cellulose; cellobiohydrolases (EC 3.2.1.91; GH5 and GH6), which catalyze the process of cellobiose release from reducing or non-reducing chain ends; and β-glucosidases (EC 3.2.1.21; GH1 and GH3), which facilitate the conversion of soluble oligosaccharides into glucose (Goyal et al. 1991; Keller et al. 2020). β-Glucosidases play a crucial role in overcoming the challenges associated with lignocellulose conversion by catalyzing the hydrolysis of cellobiose into glucose (Singhania et al. 2017). This enzymatic process not only generates fermentable sugars for bioethanol or methane production but also prevents feedback inhibition caused by cellobiose, a key intermediate in cellulose degradation that inhibits the activities of both exoglucanases and endoglucanases. By eliminating cellobiose, β-glucosidase substantially improves the overall efficiency of cellulose hydrolysis, making it essential to comprehensively understand its catalytic mechanism (Erkanli et al. 2024). In-depth knowledge of the enzymatic activity of β-glucosidase, particularly the identification of key amino acid residues involved in substrate hydrolysis, is critical for improving its efficiency.

Beyond hydrolytic enzymes, the discovery of lytic polysaccharide monooxygenases (LPMOs; AA9–AA14) redefined our understanding of fungal cellulose degradation by revealing an oxidative cleavage mechanism that operates synergistically with GHs and redox partners such as cellobiose dehydrogenases, thereby significantly improving lignocellulose depolymerization efficiency (Labourel et al. 2020). Hemicellulose degradation further requires coordinated action of xylanases (GH10 and GH11), β-xylosidases, mannanases, accessory esterases, and debranching enzymes (Xiao et al. 2024), while lignin modification relies on oxidative activities mediated by laccases (EC 1.10.3.2; CAZy AA1), lignin peroxidases (LiPs, EC 1.11.1.14, CAZy AA2), manganese-dependent peroxidases (MnPs, EC 1.11.1.13, CAZy AA2), and versatile peroxidases (VPs, EC 1.11.1.16, CAZy AA2); these enzymes are predominantly produced by white rot fungi and play a crucial role in the degradation and detoxification of lignocellulosic materials (Kumar and Chandra 2020).

Several studies have investigated microbial enzymes isolated from extreme environmental niches (Wang et al. 2024). A key strategy to predict the stability and functionality of lignocellulases involves a comprehensive characterization of these enzymes to understand their structural and functional relationships. The development of computational tools, such as NCBI BLAST for sequence alignment, ExPASy ProtParam for physicochemical profiling, Self Optimized Prediction from Multiple Alignment (SOPMA) for secondary structure prediction, SWISS-MODEL for homology modeling, and AutoDock for molecular docking, has significantly accelerated the discovery of robust enzymes for industrial applications. Currently, bioinformatic analysis has revolutionized the field of molecular biology and is essential for predicting, characterizing, and evaluating the functional properties of lignocellulases before their industrial production, through approaches such as sequence analysis and phylogeny, physicochemical property assessment, and homology modeling (Selvam et al. 2017; Poomani et al. 2024).

While several previous studies have used *in silico* tools to characterize lignocellulolytic enzymes, most of these studies have focused on few fungal species or specific enzyme types, frequently relying on commercial or model strains. In contrast, the present study reports a comprehensive multilevel computational analysis of cellulases and laccases derived from a diverse panel of fungal isolates sourced from the Moroccan environment. This work offers a broader and more functionally relevant perspective on the lignocellulosic potential of fungi. This integrative approach not only expands the catalog of uncharacterized fungal enzymes but also enhances predictive selection for downstream applications in biomass valorization. In this study, cellulases and ligninases were analyzed from fungal strains previously isolated from promising biotopes, including *Alternaria arborescens, Paecilomyces variotii, Trichocladium griseum, Penicillium chrysogenum, Penicillium crustosum, Aspergillus flavus, Fusarium solani, Penicillium citrinum*, and *Fusarium equiseti*; these fungal strains are known for their lignocellulose-degrading capabilities and have been reported as efficient biomass decomposers for sustainable applications (Arif et al. 2024). To improve our understanding of their enzymatic machinery, we conducted a comprehensive *in silico* characterization of their cellulases and ligninases.The physicochemical and structural properties of these enzymes were determined. The 3D structures of the most promising fungal enzymes were predicted by homology modeling; these structures were then docked with their specific substrates to evaluate their interaction modes and binding energies.

## Materials and methods

### Fungal strains

The fungal test strains were previously isolated from lignocellulose-rich environments in central Morocco, including decaying wood, solid byproducts of olive crushing, and their compost. All strains are filamentous fungi belonging to the phylum *Ascomycota*. A previous study (Arif et al. 2022) screened all strains for their lignocellulosic potential by conducting qualitative and quantitative assays; several other strains were further evaluated in liquid cultures containing cellulose, lignin, and olive pomace to validate their enzymatic profiles. In a subsequent work (Arif et al. 2024), one of the most promising strains from this collection was selected and cultivated under different fermentation conditions using olive stones as a local lignocellulosic substrate. Based on these results, the following nine strains with confirmed enzyme activity and the availability of lignocellulase sequences in the NCBI database were selected for *in silico* analysis: *A. arborescens* (OPP 6), *P. variotii* (OPP 1), *T. griseum* (DWB 3), *P. chrysogenum* (C 9), *P. crustosum* (DWE 4), *A. flavus* (C 13), *F. solani* (DWI 2), *P. citrinum* (DWB 2), and *F. equiseti* (STT 4).

### Retrieval of lignocellulase sequences and phylogeny construction

The sequences of cellulases and ligninases of all fungal species were retrieved from the NCBI protein database [http://www.ncbi.nlm.nih.gov]. “Fungus species” AND (“cellulase” OR “laccase” OR “lignin peroxidase” OR “manganese peroxidase”) was used as the search query. The results beyond the study’s scope were manually reviewed and filtered from the protein sequences. For each fungus species, a maximum of five sequences were randomly selected from the final list, ensuring that each sequence represents a distinct class of enzymes. Sequences were aligned using Clustal (Larkin et al. 2007) and manually filtered to exclude irrelevant results. The collected protein sequences of each enzyme group were aligned using ClustalW, and a phylogenetic tree was generated using the Neighbor-Joining method. Phylogenetic analysis was then conducted with MEGA 11 software.

### Physicochemical characterization of test enzymes and secondary structure prediction

The physicochemical properties of fungal cellulases and ligninases were analyzed *in silico* using the ProtParam tool available on the ExPASy server (Gasteiger et al. 2003, 2005). The calculated parameters included molecular weight (MW); extinction coefficient (EC), which is important for assessing the light absorption of proteins during purification processes (Gill and Hippel 1989); theoretical isoelectric point (pI); *in vivo* half-life (HL), which indicates the degradation time of synthesized proteins within cells; instability index (Ii) (Guruprasad et al. 1990); aliphatic index (Ai) (Ikai 1980); Grand Average of Hydropathy (GRAVY) (Kyte and Doolittle 1982); number of negative residues (–R); and number of positive residues (+R). Additionally, secondary structural elements were predicted using the SOPMA tool (Combet et al. 2000), which enabled the analysis of secondary conformation across different segments of the protein sequences.

### Homology modeling of 3D structure and model validation

The SWISS-MODEL server in the automated mode was used to predict the 3D structures of fungal enzymes (Schwede et al. 2003). The quality of the predicted models was validated according to the QMEAN score (Benkert et al. 2011). This process enabled the selection of the most appropriate enzyme model based on optimal identity and minimal energy interactions. The proposed models were further validated by uploading them to PROCHECK (Laskowski et al. 1993), ERRAT (Colovos and Yeates 1993), and the ProSA servers (Wiederstein and Sippl 2007).

### Molecular docking of enzyme and substrate

Molecular docking with Autodock was performed to investigate the interaction between cellobiose and four β-glucosidases. The 3D structures of β-glucosidases from *A. arborescens* RYO56859.1 (PDB ID: 3zyz), *P. variotii* GAD99276.1 (PDB ID: 4iic), *F. solani* XP_046126616.1 (PDB ID: 3ahy), and *F. equiseti* KAJ4141610.1 (PDB ID: 5nbs) were retrieved from the Protein Data Bank (PDB) and prepared following standard docking procedures. Cellobiose, obtained from PubChem (CID: 10712), was also optimized and prepared. The docking grid boxes were centered on the predicted catalytic sites of each β-glucosidase and sized to include the main residues involved in substrate recognition. Grid box centers were set as follows: 3zyz (X = 7.3511, Y = –5.2022, Z = 13.2815), 4iic (X = 40.2146, Y = –19.5251, Z = 28.1608), 3ahy (X = –7.6994, Y = –83.1419, Z = 19.6435), and 5nbs (X = 37.3259, Y = 130.1375, Z = –5.8881). To ensure the robustness of the docking results, eight independent docking runs were performed for each enzyme-substrate system using different initial random seeds. Docking solutions were evaluated based on pose convergence, binding energy, and interaction geometry. The docking protocol and comparative analysis were further validated by including β-glucosidase B (BglB) from *Paenibacillus polymyxa* (PDB ID: 2O9R) as a reference structure (Khairudin and Mazlan 2013; Mazlan and Khairudin 2014). Superimposition and RMSD analysis of the predicted poses were performed using Discovery Studio Visualizer to assess pose reproducibility and binding site consistency.

## Results and discussion

### Lignocellulase sequences and phylogenetic analyses

After eliminating putative, unknown, and unrelated enzymes and performing rigorous manual verification, 80 sequences were retrieved for detailed analysis (Table S1): 60 cellulases, mainly endoglucanases and β-glucosidases, and 20 ligninases, primarily laccases. No lignin peroxidase or manganese peroxidase sequences were detected in any of the test strains, and no lignocellulolytic enzymes were identified for *Penicillium simile* (DWE 3), *Aspergillus versicolor* (OPO 4), *Circinella muscae* (C 6), *Fusarium nygamai* (DWI 1), *Trichoderma capillare* (STT 5), *Aspergillus calidoustus* (OPO 6), *Alternaria citri* (DWE 5), *Doratomyces* sp. (C 10), and *Penicillium sizovae* (STA 3). Phylogenetic analysis was conducted on a representative subset of the sequences retrieved from the NCBI database. Distinct phylogenetic trees were generated for endoglucanases, β-glucosidases, and laccases by selecting a limited number of sequences from each species that represented the main classes of enzymes being studied.

The phylogenetic analysis of endoglucanase sequences (Figure 1A) from 7 fungal species, namely *T. griseum, F. equiseti, F. solani, A. arborescens, P. chrysogenum, P. variotii*, and *A. flavus*, revealed significant evolutionary divergence among the enzymes. The generated tree showed a well-supported clade between *T. griseum* and *F. equiseti* (bootstrap value: 100%), indicating a close evolutionary relationship between their endoglucanases. Although *F. solani* belongs to the same genus as *F. equiseti*, it occupied a separate branch, highlighting functional divergence. Similarly, species such as *P. chrysogenum* and *A. arborescens* were positioned on distinct branches, reflecting significant variations in their endoglucanase sequences. *P. variotii* and *A. flavus*, which occupied the last two branches in the tree, also showed distinct evolutionary paths, indicating differences in their endoglucanase sequences compared to those of the other species.

**Figure 1:**
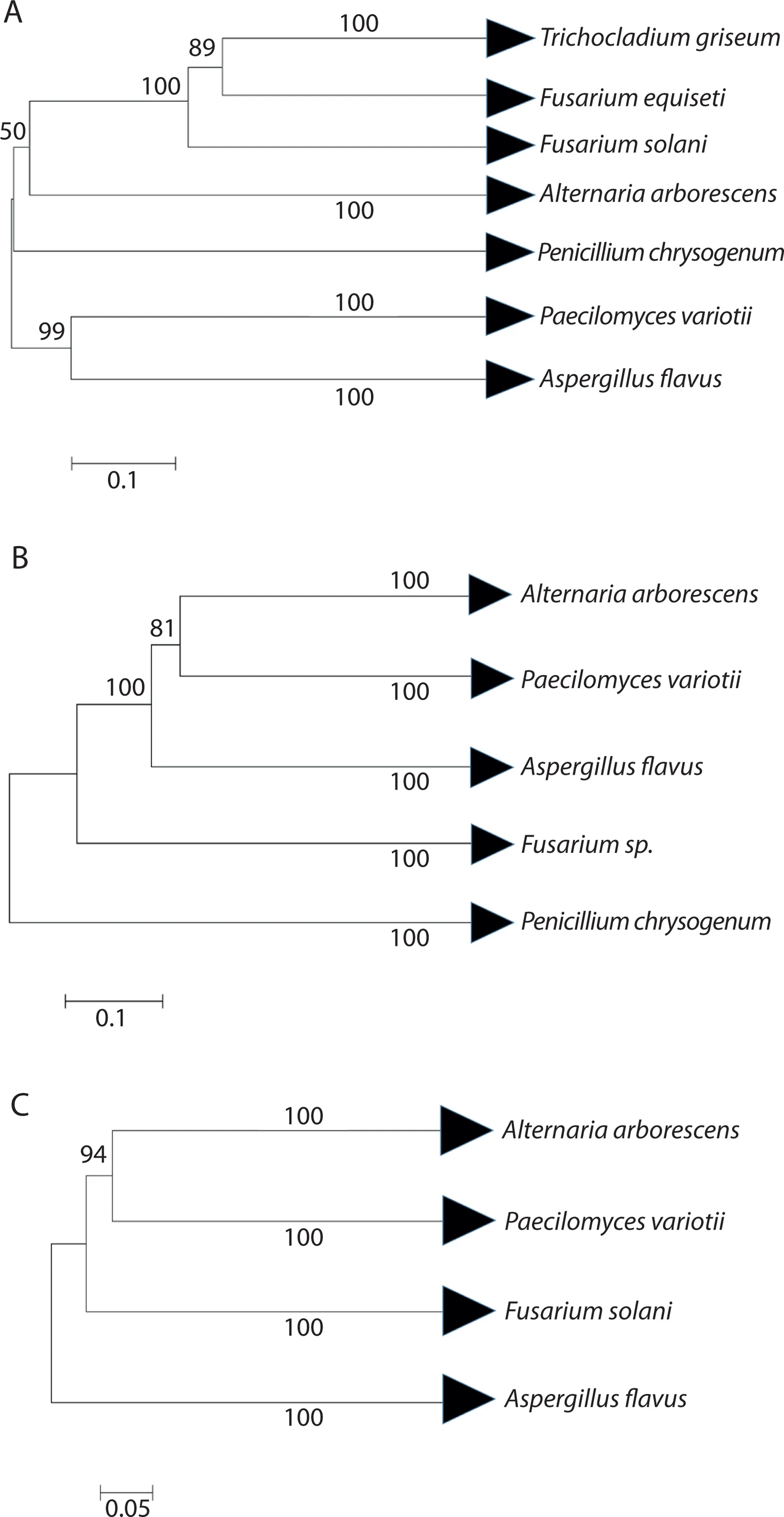
Phylogenetic analysis of sequences of (**A**) endoglucanases, (**B**) β-glucosidases, and (**C**) laccases of lignocellulolytic fungi retrieved from the NCBI database

As illustrated in Figure 1B, the phylogenetic tree for β-glucosidase enzymes displayed the evolutionary relationships among five fungal species: *A. arborescens, P. variotii, A. flavus, Fusarium* sp. (representing *F. equiseti* and *F. solani*), and *P. chrysogenum*. The tree revealed a well-supported clade between *A. arborescens* and *P. variotii*, with a bootstrap value of 100%. This finding suggests a close evolutionary relationship between their β-glucosidases, indicating that they may share functional similarities in lignocellulose degradation. Although *A. flavus* branched off separately, it had strong bootstrap support (100%), thus showing its distinct evolutionary path from the other two species, despite being grouped closer to them in this tree. At the bottom, *Fusarium* sp. and *P. chrysogenum* individually formed distinct branches, both with high bootstrap values (100%). This separation reflects the evolutionary divergence of their β-glucosidase sequences from those of the other species. The clear separation among these species highlights the diversity in their enzyme sequences, which is a key aspect for understanding how different fungi might utilize β-glucosidase in varied environmental contexts and substrates.

The phylogenetic analysis of laccase enzymes (Figure 1C) primarily focused on a representative subset of sequences rather than on the complete set of retrieved sequences to avoid generating a divergent and cluttered tree. The phylogenetic tree for laccases revealed evolutionary relationships among four fungal species: *A. arborescens, P. variotii, F. solani*, and *A. flavus*. In this tree, *A. arborescens* and *P. variotii* formed a well-supported clade with strong bootstrap support (100%), indicating a close evolutionary relationship between their laccase sequences. This finding suggests that these two species may share functional similarities in their laccase enzymes, probably playing similar roles in lignin degradation. Both *F. solani* and *A. flavus* branched off separately from the *Alternaria-Paecilomyces* clade; however, their respective branches were still highly supported, with bootstrap values of 100%. This observation indicates clear evolutionary divergence in their laccase sequences compared to the first clade, while demonstrating high confidence in the accuracy of their positioning within the tree.

The broad divergence observed across the fungal species suggests that each strain may have evolved specialized enzymatic mechanisms for degrading lignocellulose; this ability was likely influenced by their ecological niches. This variability in enzyme evolution indicates potential functional differences in lignocellulose breakdown efficiency.

### Physicochemical properties

Table S1 presents the physicochemical properties of the studied enzyme sequences, with amino acid sequence length ranging from 224 to 891 and MW ranging from 25594.92 to 96609.56 Da. Most enzyme sequences exhibited an acidic nature, except for β-glucosidase from *A. arborescens* RYO56859.1, which displayed a neutral property. Additionally, endoglucanase from *A. flavus* KAJ1709640.1 (pI 8.18) and β-glucosidase from *P. citrinum* XP_056501835.1 (pI 9.36) showed alkaline characteristics.

The results for the Ii indicated that the enzymes were stable in test tube experiments and had *in vivo* HL up to 20 h under standard conditions (Rogers et al. 1986; Tamboli et al. 2015; Buzzo et al. 2023). The latter parameter can be predicted from the Ii (Guruprasad et al. 1990). Endoglucanases from both *T. griseum* (AAM77714.2 and 1W2U_A sequences) and *F. equiseti* AAM77703.1 were very stable with an Ii value of < 20. However, the following enzyme sequences were identified as unstable (Ii ≥ 40): A0A443HK79.1 and A0A443HK11.1 from *P. variotii*; KZN90305.1 from *P. chrysogenum*; XP_056725118.1 and KAJ5394428.1 from *P. crustosum*; XP_056497110.1 and XP_056501835.1 from *P. citrinum*; and KAJ1710886.1, KAJ1705330.1, RMZ45971.1, and RAQ73286.1 from *A. flavus*.

The Ai of a protein is defined based on the aliphatic side chains of amino acids such as alanine, valine, isoleucine, and leucine. It is considered a positive factor that enhances the thermostability of globular proteins (Ikai, 1980). It may also be regarded as a positive factor for increasing the thermostability of proteins, particularly globular ones. As shown in Table S1, the following proteins had the highest Ai (>75%): *A. arborescens* β-glucosidases (XP_028505045.1, RYO56859.1, XP_028501739.1, and RYO18646.1), *A. arborescens* laccases (XP_028501087.1, XP_028510645.1, RYO69350.1, RYO39750.1, and RYO39394.1), *P. variotii* β-glucosidases (RWQ99350.1, XP_028487745.1, and GAD93537.1), *P. variotii laccases* (XP_028484177.1, RWQ94532.1, and GAD97601.1), *P. chrysogenum* endoglucanase (KZN89060.1), *P. chrysogenum* β-glucosidases (KZN90305.1, KZN93408.1, KZN93114.1, and KZN91501.1), *P. crustosum* β-glucosidases (XP_056725118.1 and KAJ5394428.1), *A. flavus* cellulases (RMZ37468.1, RAQ66523.1 and RAQ63436.1), *A. flavus* β-glucosidases (RMZ41167.1, RMZ38224.1, RMZ37309.1, RAQ79441.1, and RAQ77926.1), *A. flavus* laccases (KAJ1710886.1, KAJ1705330.1, RMZ45971.1, RMZ39576.1, and RAQ73286.1), *F. solani* β-glucosidases (KAJ4232191.1 and KAJ4211136.1), *F. solani* laccases (AGT80114.1, AHD26939.1, AHZ65142.1, and AHZ65140.1), *P. citrinum* β-glucosidases (XP_056497110.1, XP_056501835.1, XP_056501834.1, XP_056498616.1, and XP_056495445.1), and *F. equiseti* β-glucosidase (KAJ4133747.1).

All the studied enzymes had negative GRAVY index values, ranging from –0.619 to –0.001. This finding indicates better interactions between these enzymes and water. The results also showed that most lignocellulolytic proteins contained more negatively charged amino acids than positively charged ones.

### Secondary structure prediction and homology modeling of the 3D structure

The enzyme sequences of all strains exhibited optimum secondary structures (Table S1), except for β-turn, which showed poor conformation. The following predicted secondary structures also had a low content of α-helix: *A. arborescens* laccases (XP_028510645.1, RYO69350.1, and RYO39394.1), *P. variotii* endoglucanases (XP_028485664.1, RWQ96019.1, and GAD94262.1), multicopper oxidase domain-containing proteins (XP_028484177.1 and RWQ94532.1), *A. flavus* endoglucanases (KAJ1715009.1 and KAJ1709640.1), *A. flavus* laccases (KAJ1705330.1 and RMZ45971.1), and *F. solani* laccases (AGT80114.1, AHD26939.1, and AHZ65141.1). Notably, for most protein sequences, the arrangement of their different parts into random coils enhances their flexibility, providing greater conformational adaptability during enzymatic turnover (Buxbaum 2007; Tamboli et al. 2015). However, these regions are more prone to mutations and represent evolutionary hotspots (Buxbaum 2007). Based on the computed parameters, only enzyme sequences with stable physicochemical patterns and robust 2D properties were selected for further *in silico* analysis.

The 3D modeling of the selected sequences using the SWISS-MODEL server provided critical insights. Models with a QMEAN score of less than –4.0, indicating poor quality, were excluded, while lignocellulolytic enzymes with sequence identities greater than 50% were considered (Table S2). The QMEAN score estimates the overall “degree of nativeness” of the structural features in the model. It assesses whether the model’s QMEAN score aligns with the expected value from experimental structures of similar sizes. A QMEAN score close to zero indicates good agreement between the model and experimental structures. Only one enzyme sequence from similar models was selected for validation, ultimately yielding 23 lignocellulolytic enzyme sequences. Figure 2 shows an example of a homology-modeled 3D structure obtained from the SWISS-MODEL server.

**Figure 2:**
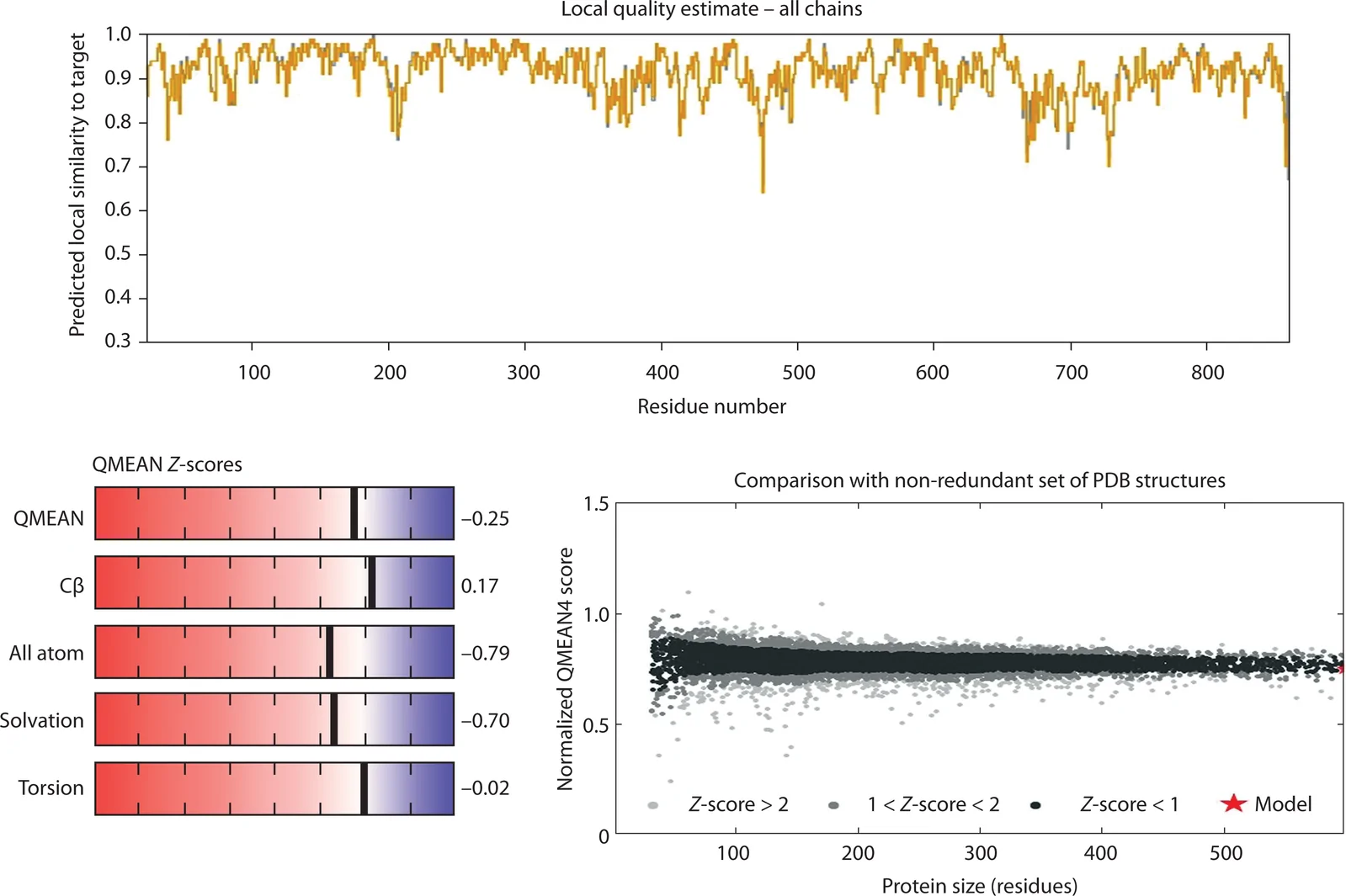
Example of homology modeling of the 3D structure result retrieved from the SWISS-MODEL server for β-glucosidase RMZ38224.1 from *Aspergillus flavus*

### Model validation and substrate docking

High-quality templates were identified based on scores of residues in the most favored region (MFR) > 90%, residues in the disallowed region (DS) < 1%, and ERRAT quality factor > 90%, reflecting good structural quality (Table S3). β-Glucosidase sequences from *A. arborescens* RYO56859.1, *P. variotii* GAD99276.1, *F. solani* XP_046126616.1, and *F. equiseti* KAJ4141610.1 were therefore considered suitable for molecular docking. Figure 3 presents the corresponding validation plots obtained from the PROCHECK, ERRAT, and ProSA servers.

**Figure 3:**
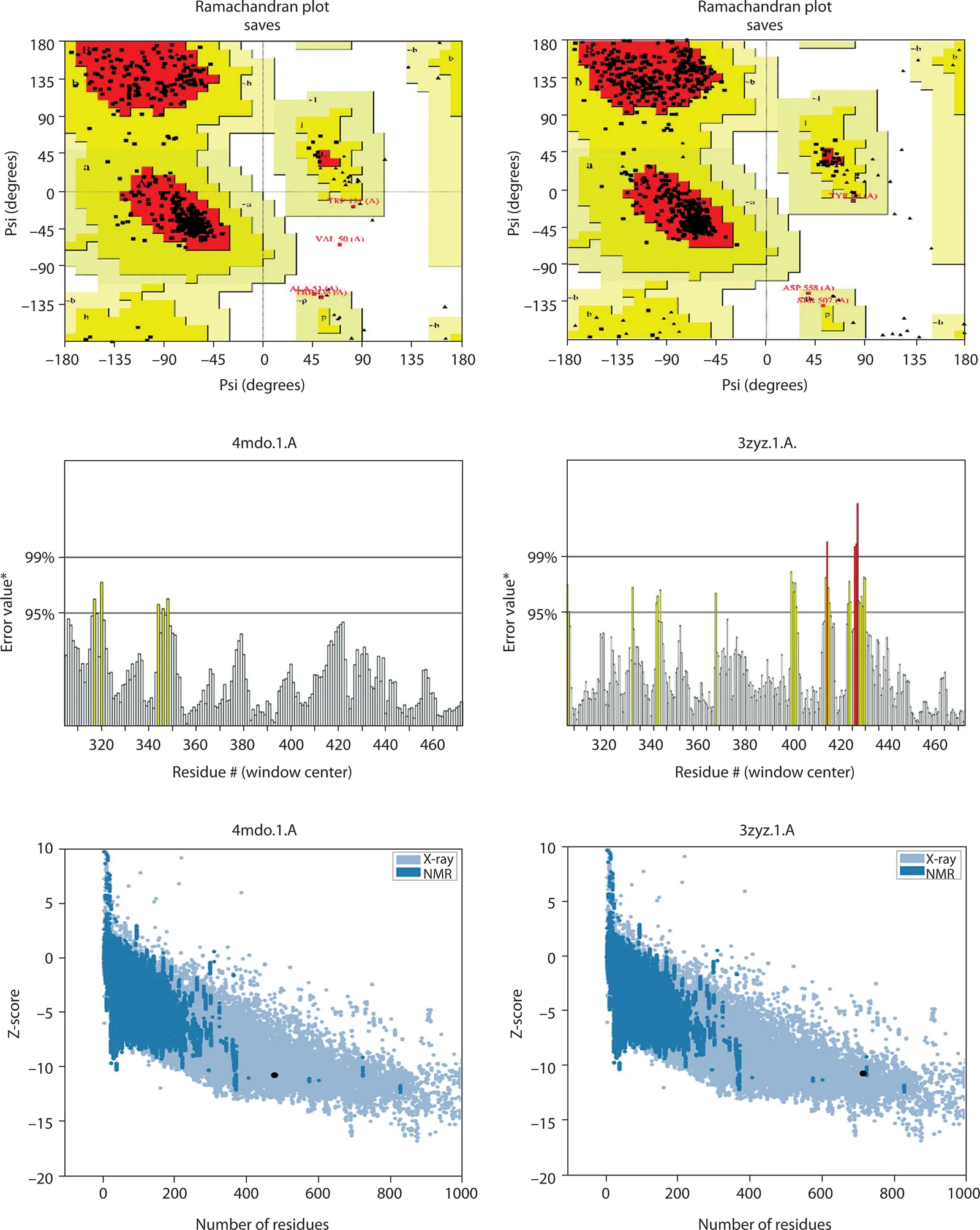
Example of graph and plots from PROCHECK, ERRAT, and ProSA servers for 4mdo.1.A and 3zyz.1.A templates

Docking of cellobiose into the four abovementioned β-glucosidases showed consistent localization of the ligand within the predicted catalytic pocket across eight independent docking runs for each enzyme. Superimposition of the top-ranked poses revealed high reproducibility, with RMSD values below 1.0 Å, supporting the robustness of the docking protocol and the stability of the predicted binding modes. The comparative docking analysis using the reference BglB from *P. polymyxa* showed that cellobiose binds within the canonical active site and adopts a binding orientation identical to that observed in the four fungal β-glucosidases analyzed in this study. The docking results revealed variations in interaction patterns, reflected by predicted binding energies and hydrogen bond networks (Table S4). Among the analyzed fungal enzymes, β-glucosidase from *F. equiseti* exhibited a favorable interaction profile with cellobiose, with a binding energy of –5.32 kcal/mol; this interaction was characterized by multiple hydrogen bonds involving Asp88, Arg94, Lys185, Gln197, Asp276, and Gln278 residues, with distances ranging from 1.95 to 2.58 Å (Figure 4D). In contrast, the β-glucosidase from *P. variotii* showed a weaker predicted interaction of -0.85 kcal/mol, despite forming multiple hydrogen bonds with residues such as Arg150 (2.18 Å), Phe161 (1.77 Å), and Asp633 (1.76 Å), suggesting a less optimal spatial complementarity within the binding pocket (Figure 4B). β-Glucosidases from *A. arborescens* and *F. solani* showed intermediate binding energies of -4.97 and -4.67 kcal/mol, respectively. For *A. arborescens*, residues Asp61 (2.08 Å), Arg67 (1.80, 2.67 Å), Arg125 (2.12 Å), Glu441 (1.69 Å), and Lys158 (2.14 Å) formed well-positioned hydrogen bonds, with additional π-σ stacking through Trp237 (3.54 Å) (Figure 4A). *F. solani* interacted through Glu165 (2.15 Å) and Asp227 (2.01 Å), with weaker auxiliary contacts involving Asn301 at 4.57 Å (Figure 4C). These variations suggest that substrate recognition mechanisms differ across fungal β-glucosidases, which is primarily reflected by interaction geometry and residue-level contacts.

**Figure 4:**
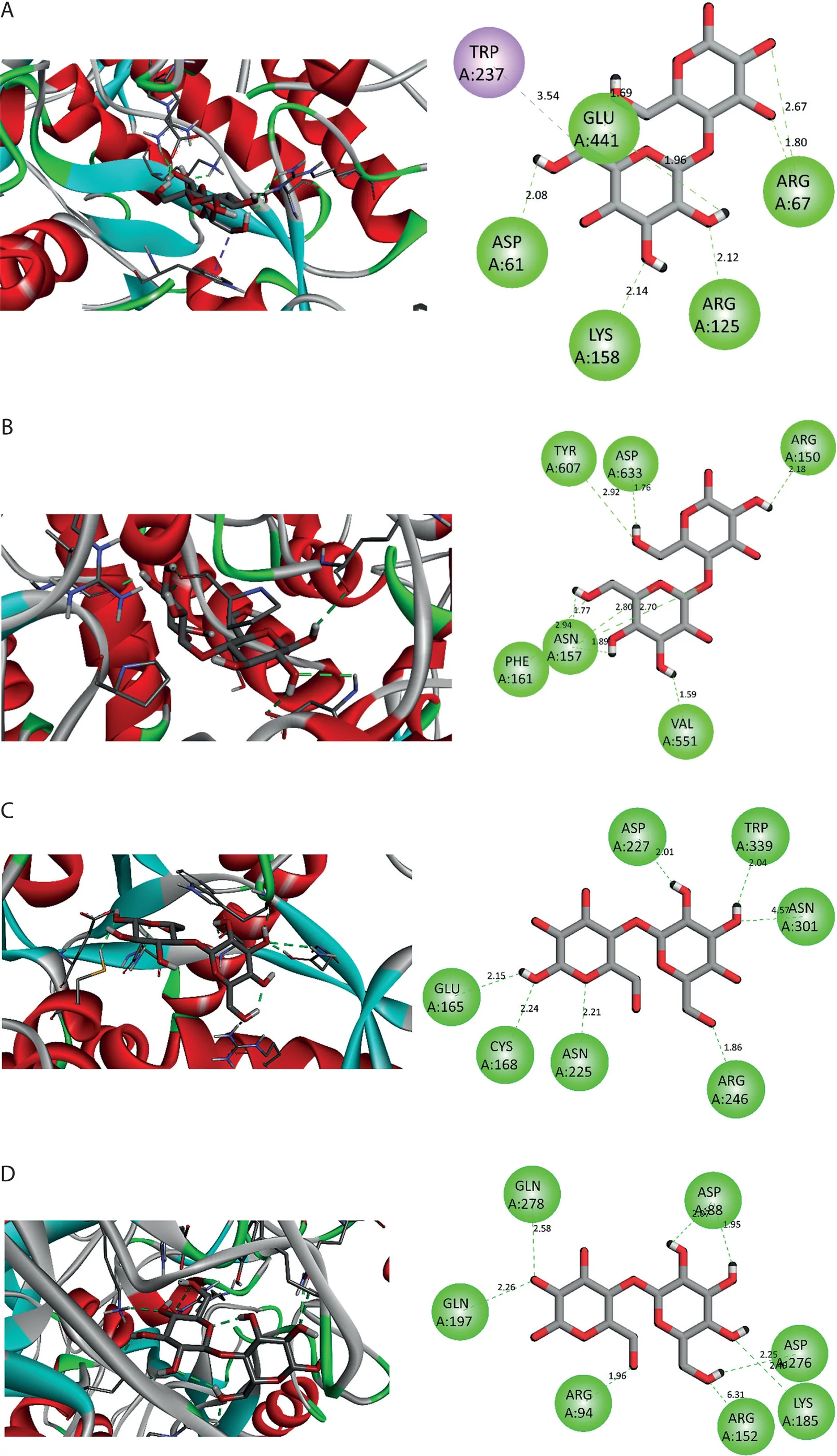
Molecular docking of (**A**) *Alternaria arborescens* (RYO56859.1), (**B**) *Paecilomyces variotii* (GAD99276.1), (**C**) *Fusarium solani* (XP_046126616.1), and (**D**) *Fusarium equiseti* (KAJ4141610.1) β-glucosidases with cellobiose as the substrate

These findings reinforce the utility of molecular docking as a predictive tool to explore enzyme-substrate interactions and identify functionally important residues (Mazlan and Khairudin 2014; Dadheech et al. 2018). Although molecular docking provides valuable insights into enzyme-ligand interactions, it merely represents a simplified approximation of enzymatic function and does not comprehensively capture *in vivo* complexity.

In particular, docking approaches typically neglect protein dynamics, solvent effects, and post-translational modifications such as glycosylation. Glycosylation can affect protein folding pathways, conformational entropy, and overall stability and can increase active site rigidity, leading to enhanced enzyme stability and catalytic turnover. Although glycan chains on the protein surface are often exposed and may not directly participate in substrate binding, their contribution to structural rigidity and robustness can indirectly affect enzymatic performance (Ramakrishnan et al. 2023; Hao et al. 2025). Consequently, the exclusion of glycosylation in the docking models may limit the interpretation of stability and dynamics-related features, even though the scope of this work was restricted to comparative substrate recognition. Future studies should focus on investigating glycosylation effects, for instance, through molecular dynamics simulations with explicit glycan modeling. Overall, the studied fungal species exhibit promising *in silico* traits that may inform their prioritization for future experimental validation in achieving lignocellulose bioconversion.

## Conclusions

This study provides a comprehensive *in silico* characterization of lignocellulolytic enzymes derived from Moroccan fungal species, highlighting their structural features and predicted substrate-binding interactions through molecular docking simulations with cellobiose. The findings reveal key active site residues and estimate the relative affinities of substrates. Despite the inherent limitations of docking-based approaches, the results suggest that the studied fungal strains may serve as valuable sources of lignocellulolytic enzymes for future research on bioconversion. Notably, β-glucosidase from *F. equiseti* exhibited the strongest predicted binding energy for cellobiose, supporting its potential as a candidate for further functional investigations, including activity assays, fermentation trials, and enzyme engineering. This work provides a computational framework that may guide enzyme selection for lignocellulose valorization. Future studies incorporating proteomic profiling, kinetic analyses, and validation under controlled fermentation conditions are critical to assess enzyme performance and industrial applicability.

## References

[cit0001] Arif S, M’Barek HN, Bekaert B, Aziz M Ben, Diouri M, Haesaert G, Hajjaj H. 2024. Lignocellulolytic Enzymes Production by Four Wild Filamentous Fungi for Olive Stones Valorization: Comparing Three Fermentation Regimens. J Microbiol Biotechnol. 34(5): 1017–1028. 10.4014/jmb.2312.12048. PMC11180923 38803105

[cit0002] Arif S, Nait M’Barek H, Oulghazi S, Audenaert K, Hajjaj H. 2022. Lignocellulose-degrading fungi newly isolated from central Morocco are potent biocatalysts for olive pomace valorization. Arch Microbiol. 204: 1–17. 10.1007/s00203-022-03318-6. 36371744

[cit0003] Barhoum A, Jeevanandam J, Rastogi A, Samyn P, Boluk Y, Dufresne A, Danquah MK, Bechelany M. 2020. Plant celluloses, hemicelluloses, lignins, and volatile oils for the synthesis of nanoparticles and nanostructured materials. Nanoscale. 12: 22845–22890. 10.1039/D0NR04795C. 33185217

[cit0004] Benkert P, Biasini M, Schwede T. 2011. Toward the estimation of the absolute quality of individual protein structure models. Bioinformatics. 27(3): 343–350. 10.1093/bioinformatics/btq662. PMC3031035 21134891

[cit0005] Buxbaum E. 2015. Fundamentals of Protein Structure and Function. Springer Cham. 10.1007/978-3-319-19920-7.

[cit0006] Buzzo BB, Giuliatti S, Pereira PAM, Gomes-Pepe ES, Lemos EG de M. 2023. Molecular Docking of Lac_CB10: Highlighting the Great Potential for Bioremediation of Recalcitrant Chemical Compounds by One Predicted Bacteroidetes CopA-Laccase. Int J Mol Sci. 24(12): 9785. 10.3390/ijms24129785. PMC10298561 37372934

[cit0007] Colovos C, Yeates TO. 1993. Verification of protein structures: Patterns of nonbonded atomic interactions. Protein Sci. 2: 1511–1519. 10.1002/pro.5560020916. PMC2142462 8401235

[cit0008] Combet C, Blanchet C, Geourjon C, Deléage G. 2000. NPS@: Network protein sequence analysis. Trends Biochem Sci. 25(3): 147–150. 10.1016/S0968-0004(99)01540-6. 10694887

[cit0009] Dadheech T, Shah R, Pandit R, Hinsu A, Chauhan PS, Jakhesara S, Kunjadiya A, Rank D, Joshi C. 2018. Cloning, molecular modeling and characterization of acidic cellulase from buffalo rumen and its applicability in saccharification of lignocellulosic biomass. Int J Biol Macromol. 113: 73–81. 10.1016/j.ijbiomac.2018.02.100. 29454942

[cit0010] Erkanli ME, El-Halabi K, Kim JR. 2024. Exploring the diversity of β-glucosidase: Classification, catalytic mechanism, molecular characteristics, kinetic models, and applications. Enzyme Microb Technol. 173: 110363. 10.1016/j.enzmictec.2023.110363. 38041879

[cit0011] Gasteiger E, Gattiker A, Hoogland C, Ivanyi I, Appel RD, Bairoch A. 2003. ExPASy: the proteomics server for in-depth protein knowledge and analysis. Nucleic Acids Res. 31(13): 3784–3788. 10.1093/nar/gkg563. PMC168970 12824418

[cit0012] Gasteiger E, Hoogland C, Gattiker A, Duvaud S, Wilkins MR, Appel RD, Bairoch A. 2005. Protein Identification and Analysis Tools on the ExPASy Server. In: Walker JM (ed.). The Proteomics Protocols Handbook. Humana Press, Totowa, NJ, pp. 571–607. 10.1385/1-59259-890-0:571.

[cit0013] Gill SC, Hippel PHV. 1989. Calculation of protein extinction coefficients from amino acid sequence data. Anal Biochem. 182: 319–326. 10.1016/0003-2697(89)90602-7. 2610349

[cit0014] Goyal A, Ghosh B, Eveleigh D. 1991. Characteristics of fungal cellulases. Bioresour Technol. 36(1): 37–50. 10.1016/0960-8524(91)90098-5.

[cit0015] Guruprasad K, Reddy BVB, Pandit MW. 1990. Correlation between stability of a protein and its dipeptide composition: a novel approach for predicting in vivo stability of a protein from its primary sequence. Protein Eng. 4(2): 155–161. 10.1093/protein/4.2.155. 2075190

[cit0016] Hao C, Zou Q, Bai X, Shi W. 2025. Effect of glycosylation on protein folding: from biological roles to chemical protein synthesis. iScience 28(6): 112605. 10.1016/j.isci.2025.112605. PMC12152670 40502693

[cit0017] Ikai A. 1980. Thermostability and Aliphatic Index of Globular Proteins. J Biochem. 88(6): 1895–1898. 7462208

[cit0018] Jahangeer M, Ur M, Rubina R, Muhammad N, Bushra N. 2025. Biotransformation of Lignocellulosic Biomass to Value-Added Bioproducts: Insights into Bio-Saccharification Strategies and Potential Concerns. Top Catal. 68: 929–950. 10.1007/s11244-024-01941-9.

[cit0019] Keller MB, Sørensen TH, Krogh KBRM. et al. 2020. Activity of fungal β-glucosidases on cellulose. Biotechnol Biofuels. 13: 121. 10.1186/s13068-020-01762-4. PMC7350674 32670408

[cit0020] Khairudin NBA, Mazlan NSF. 2013. Molecular docking study of beta-glucosidase with cellobiose, cellotetraose and cellotetriose. Bioinformation. 9(16): 813–817. 10.6026/97320630009813. PMC3796882 24143051

[cit0021] Kumar A, Chandra R. 2020. Ligninolytic enzymes and its mechanisms for degradation of lignocellulosic waste in environment. Heliyon. 6(2): e03170. 10.1016/j.heliyon.2020.e03170. PMC7033530 32095645

[cit0022] Kyte J, Doolittle RF. 1982. A simple method for displaying the hydropathic character of a protein. J Mol Biol. 157(1): 105–132. 10.1016/0022-2836(82)90515-0. 7108955

[cit0023] Labourel A, Frandsen KEH, Zhang F, et al. 2020. A fungal family of lytic polysaccharide monooxygenase-like copper proteins. Nat Chem Biol. 16: 345–350. 10.1038/s41589-019-0438-8. 31932718

[cit0024] Larkin MA, Blackshields G, Brown NP, Chenna R, Mcgettigan PA, Mcwilliam H, Valentin F, Wallace IM, Wilm A, Lopez R, et al. 2007. Clustal W and Clustal X version 2.0. Bioinformatics. 23(21): 2947–2948. 10.1093/bioinformatics/btm404. 17846036

[cit0025] Laskowski RA, MacArthur MW, Moss DS, Thornton JM. 1993. PROCHECK: a program to check the stereochemical quality of protein structures. J Appl Crystallogr. 26(2): 283–291. 10.1107/S0021889892009944.

[cit0026] Lombard V, Ramulu HG, Drula E, Coutinho MP, Henrissat B. 2014. The carbohydrate-active enzymes database (CAZy) in 2013. Nucleic Acids Res. 42: D490–D495. 10.1093/nar/gkt1178. PMC3965031 24270786

[cit0027] Mazlan NSF, Khairudin NBA. 2014. Docking Study of β-glucosidase B (BglB) from P. *polymyxca* with Cellobiose and Cellotetrose. J Med Bioeng. 3(2): 78–83.

[cit0028] Poomani MS, James SB, Muthan K, Subramanian V. 2024. Unravelling yeast cellulase potential: a computational approach to structural study, cellulolytic activity, and docking. J Mol Struct. 1300: 137254. 10.1016/j.molstruc.2023.137254.

[cit0029] Ramakrishnan K, Johnson RL, Winter SD, Worthy HL, Thomas C, Humer DC, Spadiut O, Hindson SH, Wells S, Barratt AH, Menzies GE, Pudney CR, Jones DD. 2023. Glycosylation increases active site rigidity leading to improved enzyme stability and turnover. FEBS Journal 290(15): 3812–3827. 10.1111/febs.16783. PMC10952495 37004154

[cit0030] Rogers S, Wells R, Rechsteiner M. 1860. Acid Sequences Common to Rapidly Degraded Proteins: The PEST Hypothesis. Science. 234(4774): 364–648. 10.1126/science. 2876518

[cit0031] Schwede T, Kopp J, Guex N, Peitsch MC. 2003. SWISSMODEL: An automated protein homology-modeling server. Nucleic Acids Res. 31(13): 3381–3385. 10.1093/nar/gkg520. PMC168927 12824332

[cit0032] Selvam K, Senbagam D, Selvankumar T, Sudhakar C, Senthilkumar B, Govarthanan M. 2017. Cellulase enzyme: homology modeling, binding site identification and molecular docking. J Mol Struct. 1150: 61–67. 10.1016/j.molstruc.2017.08.067.

[cit0033] Singhania RR, Patel AK, Saini R, Pandey A. 2017. Industrial Enzymes: β-Glucosidases. Curr Dev Biotechnol Bioeng. 103–125. 10.1016/B978-0-444-63662-1.00005-1.

[cit0034] Tamboli AS, Rane NR, Patil SM, Biradar SP. 2015. Physicochemical characterization, structural analysis and homology modeling of bacterial and fungal laccases using in silico methods. Netw Model Anal Health Inform Bioinforma. 4: 17. 10.1007/s13721-015-0089-y.

[cit0035] Wang Y, Jiao M, Zhao Z, Wang Y, Li T, Wei Y, Li R, Yag F. 2024. Insight into the role of niche concept in deciphering the ecological drivers of MPs-associated bacterial communities in mangrove forest. Water Res. 249: 120995. 10.1016/j.watres.2023.120995. 38071907

[cit0036] Wiederstein M, Sippl MJ. 2007. ProSA-web: Interactive web service for the recognition of errors in three-dimensional structures of proteins. Nucleic Acids Res. 35. 10.1093/nar/gkm290. PMC1933241 17517781

[cit0037] Xiao M, Liu YJ, Bayer EA, Kosugi A, Cui Q, Feng Y. 2024. Cellulosomal hemicellulases: Indispensable players for ensuring effective lignocellulose bioconversion. Green Carbon. 2(1): 57–69. 10.1016/j.greenca.2024.01.003.

